# Chicago Pathological Society

**Published:** 1888-12

**Authors:** 


### SOCIETY REPORTS.

### CHICAGO PATHOLOGICAL SOCIETY.

Stated meeting, November 12, first vice-president, John D. Skeer, M. D., in the chair.

The committee, composed of Drs. A. E. Hoadley, C. D. Wescott, and L. B. Hayman, appointed to report upon Professor Danforth's case of gallstones, submitted the following: With the meagre material at our disposal, from which we were expected to report on the etiology, pathology, and rational treatment of the patient, we found ourselves in the pos'tion of the naturalist who attempts to define the character and habits of an extinct animal by studying one of his fossil bones. The pathological specimen submitted for our inspection consisted of a gall bladder, right and left hepatic ducts, a small portion of the common bile-duct, and a small portion of liver from the left and right lobes.

On examination we found the following conditions :

1. The portion of the common duct attached to the specimen was about two inches in length, somewhat bound down, and firmly adherent to its peritoneal covering. There was an evident increase of the connective tissue, which was firm and inelastic. The lumen of the tube would readily admit the index finger. The walls were thick, firm, and smooth, calibre uniform, and no pouches to indicate that it had ever been occupied by stones.

2. The gall bladder was about one-fourth the normal size and almost completely buried in liver tissue by the contractions of old inflammatory adhesions. Its cavity was occupied by three large biliary calculi. The duct of the gall bladder was enlarged, firmly adherent to the peritoneum and liver tissue, and communicated freely with the common duct.

3. The right hepatic duct was enlarged, the walls thickened; the first branches were also dilated and thickened, but no evidence of rupture within the liver tissue. It freely communicated with the common duct.

4. The left hepatic duct, with its branches, was very much dilated and thickened. The branches, two or more, had ruptured within the substance of the left lobe of the liver. The laceration of the liver substance extended to the peritoneum and its under surface.

The foregoing morbid changes, excepting the laceration, appear to have existed for a long time, and, taken together with the fact that some seven or eight years ago the patient suffered an acute attack, we conclude that the trouble must date from that time and progressed in the following manner as near as we can judge from the history of the case. We know that there was an obstruction to the common duct at or near the duodenum. As there was no evidence of obstruction in that part of the tube still attached to the specimen, we conclude that the stenosis was the result of an inflammation, and that the inflammation commenced at or near the seat of stricture, extended to the peritoneum, producing a localized peritonitis involving the visceral layer in the region from which the specimen was taken and resolution was established. How much more extensive the inflammation was we can not say. We conclude, however, that this inflammation occurred as the primary trouble seven or eight years ago. Since the occurrence of this inflammation the patient had not been well. She suffered repeated attacks of pain, nausea, and jaundice, with all the symptoms attendant upon an obstruction to the flow of bile. But it was not until the commencement of the present year that the obstruction was made complete, whether by the gradual contraction of the products of a previous inflammation, or by a recent inflammation, or the lodgment of a calculus, can not be known. After the complete closure of the common duct the accumulated bile was not provided for in the usual way, by the rapid distention of the gall bladder, as this viscus was bound by inflammatory adhesions, and the tubes previously thickened would not yield. The absorption and elimination by the kidneys was not sufficient to relieve the tension. Finally, the liver itself began to yield in the substance of the left lobe. There was a laceration that reached the under surface against the peitoneum. Here a bile-cyst was formed, which protruded from beneath the left lobe and in contact with the anterior abdominal wall, where it became adherent. It was tense, painful, and exquisitely sensitive. The patient's condition was one of *extremis*.

It was at this time that Professor Danforth first saw the case. Under the circumstances he made a diagnosis. He recognized that the contents of this tumor were fluid, and that the fluid was bile, and that there were also present gallstones. This remarkable diagnosis was verified by the operation. The operation was incomplete. To have completed the operation would have been to restore the continuity of the lumen of the common bile-duct with that of the intestine, directly or indirectly, by making an artificial opening between the gall bladder and an available loop of intestine lower down. The time for completing the operation, however, never came; the patient could not rally. From the careful study of the very perfect record of the case subsequent to the operation, your committee is of the opinion that nothing could have been done that was not. But prior to the operation, from January, 1888, when Dr. C. M. Weems, of Baylis, Illinois, diagnosed obstruction to the flow of bile, the case was ripe for operation. The idea that it was malignant had taken possession of the attending surgeons, and the operation was delayed so long that it was never finished. The history of this one case is sufficient to justify an exploratory incision in all similar cases where there is any doubt.

As far as the literature is concerned, cases of this kind are rare. Dr. H. M. Biggs (*The Medical Record*, November 10, 1888) presented a specimen of multiple gallstones in the liver to the New York Pathological Society, removed from the body of a man who had died in Bellevue Hospital. No history of the case had been obtainable. At the autopsy were found evidences of extensive fibro-serous peritonitis; there were also pleural adhesions and acute pericarditis. All through the liver the smaller bile-ducts were dilated, some forming cysts of considerable size and containing gallstones. Small abscesses had formed on the upper surface of the liver, and there were adhesions between this viscus and the diaphragm. There was one abscess in the left lobe, three centimetres in diameter, which, like the cysts, contained gallstones. There was also a smaller abscess in the right lobe. From one of these abscesses the inflammatory process had extended into the pericardium, and from the other into the pleural cavity. The gall bladder contained no calculi and the larger ducts were also free from them.

Professor C. W. EARLE said he was called in consultation with Dr. Denslow Lewis, two or three months ago, to see a case of gallstones. The patient, a lady, had suffered from several attacks of jaundice from time to time, and on admission into the Cook County Hospital was greatly emaciated. Dr. Lewis, deeming an operation justifiable, made the usual incisions and removed 183 gallstones, by absolute count, most of them being about the size of a small pea, and three about the size of the tip of the little finger. The patient remained in the hospital a month after the operation, and was discharged, having made an excellent recovery.

Professor MARIE J. MERGLER read a paper, entitled "Report of a Case of Placenta Prævia Marginata."

During the winter of 1887–8, Mrs. O—— engaged me for her confinement, which was expected about July 1.

*Physical Condition.*—Brunette, æt. 39, very pale, medium stature, weight about 140, lower extremities somewhat œdematous, quite varicose. Appetite fair; bowels inclined to constipation; urine normal.

*Nervous Condition.*—More excitable than normal.

The patient had felt an unusual solicitude about her condition. She had recently attended her mother's funeral, and had subsequently dreamt that she would die during confinement. Being naturally somewhat superstitious, she attached great importance to this dream, and looked upon it as a very bad omen.

Her previous labors (9) had been normal, but unusually rapid. May 17, she awoke in the night and found that a severe uterine hæmorrhage had taken place, which, however, soon ceased spontaneously. Early the next morning I was called in. I found her looking very pale, and feeling much exhausted. She stated that the hæmorrhage came on without uterine pain or perceptible contractions, but that on the day previous she had had little more backache than usual.

On examination the cervix presented an uneven surface, like that of a mass of corrugated vessels. This soft body extended beyond the os on all sides, and no presenting part could be distinguished. The internal os was closed. I made the diagnosis *placenta prævia centralis.* She had slight attacks of syncope, for which I lowered the head and ordered half-ounce doses of wine. There was but slight hæmorrhage. I washed the vagina with carbolized water and packed it with strips of iodoform gauze, and advised the family to adopt every means of securing perfect mental quietude for the patient, at the same time charging them to notify me of the first indication of hæmorrhage or pain. On the second day the packing was removed. There had been neither hæmorrhage nor pain. She kept her bed for a week, when another hæmorrhage occurred. This time, the internal os was opened sufficiently to admit the index finger. The presenting head could be distinctly felt, with no placental structure interfering, except on the right side where the edge of the placenta presented.

Diagnosis was now changed to *placenta prævia marginalis.* As she was continually threatened with attacks of fainting, I advised immediate delivery, but requested to have a consultant in the case. To this the husband consented, and I called in Professor Earle, who, on examining the patient, concurred in the diagnosis, and also advised immediate delivery.

The vagina was packed with iodoform gauze for twenty-four hours. On removal of this there was but slight hæmorrhage. The os was but little dilated and rigid, and no presenting part could be distinctly felt. At 8 p. m., I removed the packing and remained with the patient. She slept during the greater part of the night until 2 a. m., when she felt strong contractions. There was slight hæmorrhage.

On removal of the packing the os was found well dilated and the head could again be felt, but was easily displaced. I sent for Professor Earle, but as hæmorrhage increased and the head would not engage, I at once ruptured the membranes and brought down the foot, which was easily accomplished, on account of the great amount of amniotic fluid and great mobility of the fœtus. The hæmorrhage ceased and the patient had fair pains. On Professor Earle's arrival, the expulsion was much assisted by firm pressure upon the fundus. Labor was completed in about three-quarters of an hour. The placenta was slightly adherent and easily removed. No anæsthetic was used. The fœtus, which was poorly developed, expired soon after birth. She lost but little more blood than in an ordinary labor, but considerable stimulation was required to ward off the attacks of fainting. After the administration of an intrauterine bichloride douche, the uterus contracted firmly. During convalescence the excessive anæmia was the only alarming symptom. The uterus remained well contracted; temperature, 98½° and 99° Fahr., except on the third day, when her breasts were quite painful, the temperature rising to 100°. On the following day and thereafter it was normal. She regained her usual degree of health in six weeks.

Professor H. M. LYMAN had had two or three cases during the last twenty-five years. When hæmorrhage comes on it is his rule to deliver as speedily as possible, his aim always being to protect the mother without regard to the consequences to the child. The salvation of the mother is a thing of prime importance.

Dr. A. B. STRONG had only met with one case in his practice in which he got the afterbirth away first. There was profuse bleeding before delivery, but as soon as the placenta had been removed the hæmorrhage ceased.

Dr. C. D. WESCOTT: A thought has occurred to me as to rupturing through a placenta attached centrally. I would like to ask: Can we do that and deliver with safety to the child? I have met with but one case of placenta prævia, and in that there had been three quite copious hæmorrhages before labor commenced, and the case convinced me that if ever I see any more, and I hope I shall not, I shall induce labor at once, if the family and my consultant will consent. The case that I refer to was one of marginal attachment, with laceration of the border of the placenta. The placenta was quite firmly adherent. I tried to detach the margin to stop the hæmorrhage, and succeeded partially, but I could demonstrate by the touch that most of the hæmorrhage came from the tear in the edge of the placenta. I could not determine positively whether the child was alive or not at the beginning of labor, for I had no stethoscope; it was certainly not alive when labor was completed. I introduced my hand, as soon as dilatation had advanced far enough to permit it, and had no difficulty in bringing down one of the legs through the os uteri in the manner described by Dr. Earle. Having no evidence that the child was alive, I did not hasten delivery, consequently an hour or more was consumed in the operation. The child had the appearance of being exsanguinated, and, though efforts at resucitation were made, no sign of life was manifested. The placenta was removed with some difficulty, but the mother made a good recovery.

Professor A. E. HOADLEY: The remarks of the previous speakers have induced me to say a word on this paper, and I desire in the first place to commend the position taken in the case. I hold that we are perfectly justified in inducing early labor in these cases. But there is one point that occurs to me that has not been touched upon by Professor Earle, and that is, with regard to the child's condition. In placenta prævia it is not only the mother that bleeds, but the child also. We know that the young infant at birth can not tolerate the loss of blood well. If there have been two or three hæmorrhages, for example, and, as a consequence, a certain degree of anæmia of the mother, there should be, and usually is, an alarming condition of the infant. I have had three cases of placenta prævia, and all the babies were born alive, but died soon after birth. One of these cases had had only a second hæmorrhage. The first hæmorrhage stopped spontaneously, and with good care the mother went to nearly full term. The child was delivered alive, but soon expired.

Where placenta prævia is made out to be the cause of hæmorrhage, both mother and child are losing blood, and I think immediate delivery is justifiable.

A CASE OF CHYLURIA.—Professor HOADLEY reported a case of chyluria, and said: I have here a sample of urine which came from a chyluric patient. In addition to the normal ingredients, it has about 1 per centum of fat in emulsion. Chyluria occurs in two forms, first, the sporadic, and, second, a form of chyluria that is dependent upon a worm that infests the human blood, and is prevalent in the tropical regions, called *filaria sanguinis*. I have examined this patient for filariæ, but have found none, and must regard it as a case of sporadic chyluria. The patient is a young man twenty-two years of age, of excellent habits, and enjoys good health. He has no symptoms that could lead us to believe that there was any organic change anywhere. Examination of the urine by the microscope furnishes no evidence. He passes clear urine in the evening. During the middle of the day he will pass many coagula that are sometimes translucent, and at other times milky white.

The pathology of these sporadic cases is unknown. There are several theories. One is, that there are communications existing between the lympathic system and the urinary system somewhere; that the lymphatic vessels dilate until they reach some of the lymphatic vessels that communicate with the substance of the kidney: and in this way let fat infiltrate into the kidney. Chemical analysis shows that that could not be the case, inasmuch as in normal chyle there is more fat, and there are other elements that are not found in this chylous urine. I have an idea that it is a fault in digestion, not alimentary digestion, but the digestion that takes place somewhere between the time that the chyle containing emulsified fat is poured into the blood, and where the blood comes to nourish the tissues. A change takes place in the blood after the chyle is poured into the venous circulation that converts the fat into its primitive elements. How that particular process is formed I am not prepared to say, but it is arrested and the fat is eliminated by the kidneys.

Chyluria can be artificially produced by injecting oil into the blood; the kidneys will eliminate it.

The remedies I have given have had no effect. I have given ergot, atropine, strychnine separately and thoroughly without any beneficial results. The only abnormality is the presence of fat.

Professor Lyman asked whether the patient had been examined at different times of the day and night.

Professor Hoadley: Filaria are never found in the blood except at night. I have examined the patient at night four different times without being successful in finding them; also examined him in the day time.

